# Food deprivation differentially modulates gene expression of LPXRFa and kisspeptin systems in the brain-pituitary axis of half-smooth tongue sole (*Cynoglossus semilaevis*)

**DOI:** 10.3389/fendo.2023.1099832

**Published:** 2023-03-24

**Authors:** Bin Wang, Aijun Cui, Yongjiang Xu, Yaxing Zhang, Yan Jiang, Xuezhou Liu

**Affiliations:** ^1^ Key Laboratory of Sustainable Development of Marine Fisheries, Ministry of Agriculture and Rural Affairs, Yellow Sea Fisheries Research Institute, Chinese Academy of Fishery Sciences, Qingdao, China; ^2^ Joint Laboratory for Deep Blue Fishery Engineering, Pilot National Laboratory for Marine Science and Technology (Qingdao), Qingdao, China

**Keywords:** LPXRFa, LPXRFa receptor, kisspeptin, kisspeptin receptor, *Cynoglossus semilaevis*

## Abstract

LPXRFa, also known as gonadotropin-inhibitory hormone (GnIH), and kisspeptin (Kiss) are two major hypothalamic peptides that modulate the reproductive axis of vertebrates, including teleosts. However, little information is available regarding the actions of nutritional status on the regulation of these two neuroendocrine systems in fish. Herein, we assessed the effects of starvation and refeeding on the expression of *lpxrfa*, *kiss2* and their receptors (*lpxrfa-r* and *kiss2r* respectively) at the brain-pituitary level of half-smooth tongue sole (*Cynoglossus semilaevis*). Food deprivation for 4 weeks induced a rise in brain *lpxrfa* as well as brain and pituitary *lpxrfa-r* mRNA levels, and refeeding restored brain *lpxrfa* and *lpxrfa-r* expression back to normal. However, pituitary *lpxrfa-r* mRNA levels still remained high after 1 week of refeeding. Neither *lpxrfa* nor *kiss2* transcripts in the pituitary were altered by fasting, but their mRNA levels increased significantly after 1 week of refeeding, and declined back to the control levels after 2 weeks of refeeding. None of brain *kiss2* and *kiss2r* along with pituitary *kiss2r* transcripts were modified by the nutritional status. In summary, our results revealed an interaction between energy status and the elements of LPXRFa and Kiss systems in the brain-pituitary axis of half-smooth tongue sole. Food deprivation and refeeding differentially regulated the two systems, which provided additional evidence for the involvement of the LPXRFa and Kiss systems in the regulation of reproduction by energy balance in non-mammalian species.

## 1 Introduction

In 2000, a novel hypothalamic neuropeptide was discovered in the Japanese quail, and was termed gonadotropin-inhibitory hormone (GnIH) based on its ability to inhibit gonadotropin release (1). Subsequently, its homologues have been identified in various vertebrates, including fish, amphibians, reptiles, birds and mammals (2–4). GnIH is also called LPXRFa in teleosts or RFamide-related peptide (RFRP) in mammals, and its precursor encompasses two, three or four putative/mature peptides depending on the species, which generally possess a common C-terminal LPXRFamide (X = L or Q) motif (3–5). There is compelling evidence that GnIH exerts an inhibitory effect on each level of the brain-pituitary-gonadal axis *via* its cognate receptor GPR147 (namely GnIH-R or LPXRFa-R), and it also participates in stress response, biological rhythms and social behaviors (6–8). Three different GPR147 types are found in some Cypriniform species, but only one exists in other vertebrates investigated so far (4). The molecular mechanisms of GnIH actions have been investigated in mammals (9–12), chicken (13), Nile tilapia (14), orange-spotted grouper (15), zebrafish (16), half-smooth tongue sole (17), chub mackerel (18), and European sea bass (19). It is of note that activation of GnIH receptor can interfere with signaling pathways induced by other neuroendocrine factors (9–13, 16, 19–21).

Following the discovery of GnIH, another hypothalamic neuropeptide kisspeptin (Kiss) has been recognized as an essential stimulator of reproduction in mammals (22, 23). In contrast to most mammals in which only one *kiss* and one receptor genes have been characterized, up to three *kiss* genes (*kiss1*, *kiss2* and *kiss3*) and four receptor genes (*kissr1*, *kissr2*, *kissr3* and *kissr4*) have been identified in non-mammalian species (24), which increases the complexity of the Kiss/KissR systems involved in the control of reproduction (25, 26). In most teleost species, both *kiss1* and *kiss2* along with *kissr2* and *kissr3* genes have been reported, whereas only the *kiss2*/*kissr2* system was identified in other fish species, including half-smooth tongue sole (26, 27). There is considerable evidence supporting that Kiss exerts a stimulatory action on teleost reproduction, as in mammals (26–28). However, recent studies on gene knockout of *kiss* and/or *kissr* in zebrafish and medaka revealed that the Kiss/KissR system is dispensable for normal reproduction (29–32). Thus, much more mutant studies in various fish species are still required to clarify the reproductive role of the Kiss/KissR system in teleost.

A close association between energy balance and reproduction has been documented, and various hypothalamic neuropeptides are involved in the regulation of these two critical physiological processes, either directly or indirectly (33, 34). For example, LPXRFa stimulated food intake in chicks (35), Pekin ducks (36), sheep (37), mice (37), rats (38), jerboa (39), and cynomolgus monkeys (37). Conversely, Kiss reduced appetite in rats (40), mice (41, 42) and jerboa (39). Such comparative studies have not yet been performed in teleosts. In addition, hypothalamic *lpxrfa* mRNA levels were increased during depressed food intake in heat-exposed chicks (43). However, food deprivation resulted in a decrease in the number of LPXRFa-immunoreactive neurons in the hypothalamus of a female songbird, the zebra finch (44). Fasting had no effect on the number of hypothalamic LPXRFa-immunoreactive cell bodies or *lpxrfa* mRNA expression in zebra finch males (45). Gonadal *lpxrfa* mRNA levels did not differ between fasted and control males, either (45). Similarly, no changes in hypothalamic *lpxrfa* transcripts were noticed in hens maintained on a diet restricted to 50% of ad libitum feeding, compared to control hens allowed free access to food for 7 days (46). To the best of our knowledge, only one report is available in fish regarding the effect of feeding status on the LPXRFa/LPXRFa-R system (47), in which fasting increased *lpxrfa* mRNA levels in the brains of wild-type zebrafish females and Casper zebrafish males, respectively.

In addition, Kiss has emerged as a molecular switch between reproduction and energy homeostasis in vertebrates. Fasting induced a decline in *kiss1* and *kissr1* mRNA levels in the hypothalamus of mice (48). Interestingly, food deprivation led to a concomitant increase in hypothalamic *kissr1* and decrease in *kiss1* mRNA levels in prepubertal rats (49). On the contrary, starvation stimulated hypothalamic mRNA levels of *kiss2* and *kissr2* in Senegalese sole (*Solea senegalensis*) (50), and up-regulated *kiss2* and *kissr2_v1* expression in the hypothalamus of pejerrey (*Odontesthes bonariensis*) (51). Taken together, the molecular mechanisms mediating the effects of negative energy balance on reproduction may differ among various species, which merits further studies (26, 52).

Using the half-smooth tongue sole (*Cynoglossus semilaevis*) as a model, we have previously cloned the full-length cDNA sequences of *lpxrfa*, *lpxrfa*-*r*, *kiss2* and *kiss2r* (also called *kissr2*), and provided evidence for their implication in the control of reproduction and the possible signaling pathways elicited by LPXRFa and Kiss2 peptides as well as their interaction on cell signaling (17, 20, 21, 53–56). Given that the way energy balance affects the reproductive axis is still poorly understood in fish, this study aimed to evaluate the effects of nutritional status on the transcript levels of both LPXRFa/LPXRFa-R and Kiss2/Kiss2R systems at the brain-pituitary levels of half-smooth tongue sole.

## 2 Materials and methods

### 2.1 Animals

Approximately 2-year-old female half-smooth tongue soles were purchased from a local fishery (Qingdao, China), and maintained in an indoor concrete tank with recirculating seawater (dissolved oxygen > 5 mg/L, pH 7.8–8.2, salinity 27–31 ppt, and water temperature 24–26°C). Fish were exposed to a cyclical photoperiod (12L:12D) and fed to satiation twice daily as described in detail previously (57).

### 2.2 Starvation and refeeding experiment

In order to investigate the effects of nutritional status on mRNA levels of *lpxrfa*, *kiss2* and their receptors, we compared two groups of half-smooth tongue sole, one under normal feeding condition, and the other submitted to starvation followed by refeeding. The experiment was previously performed (57) where half-smooth tongue sole females with an average body weight of 530 g were divided into two groups: one (control group) was fed to satiation twice daily as mentioned above, and the other (starved group) was fasted for 4 weeks and then refed to satiation twice daily for 2 weeks. Brain and pituitary were collected from each group at 2, 4, 5 and 6 weeks, respectively, frozen in liquid nitrogen and stored in -80°C freezer. The same cDNA samples, which were used to detect *spx2* gene in our previous study (57), were used to analyze *lpxrfa*, *lpxrfa-r*, *kiss2*, and *kiss2r* mRNA levels in the present study.

### 2.3 RNA isolation and RT-qPCR assay

Total RNA from the brain and pituitary was isolated and reverse transcribed to cDNAs which were used as templates for qPCR analysis of *lpxrfa*, *lpxrfa-r*, *kiss2* and *kiss2r* in this study. The PCR amplification was carried out on Mastercycler^®^ ep *realplex* Real-time PCR System (Eppendorf), and the thermal cycling parameters were as follows: 95°C for 30 s, and 40 cycles of 95°C for 5 s and 60°C for 20 s. Data were calculated by the comparative Ct method using *18s* as a reference gene (20). The specific primers and amplification size values for each gene are shown in **Table 1**.

**Table 1:** List of primers used in this study.

Primer name	Primer sequence (5'-3')	Amplicon size (bp)	GenBank accession No.
*lpxrfa*-F	GGAAATCAGCCTACAGTGACAAAA	120	KU612223
*lpxrfa*-R	GCCTCTCCAAGTCCAAACTCC		
*lpxrfar*-F	GCTTTTCATGTTGTCCTGGTTG	147	KX839491
*lpxrfar*-R	GGGTTGATGCTTGAGTTGGAG		
*kiss2*-F	GGCAACTGCTGTGCAACGA	133	KX090946
*kiss2*-R	AAGACAGAAAGCGGGGAGAAC		
*kiss2r*-F	AGTTGTGATCGTCCTCCTCTTTG	92	KX685668
*kiss2r*-R	AGTTGGGTTGGTATTTGGGATG		
*18s* F	GGTCTGTGATGCCCTTAGATGTC	107	GQ426786
*18s* R	AGTGGGGTTCAGCGGGTTAC		

### 2.4 Statistical analysis

The results were analyzed by Student’s t-test using SPSS17.0, and are presented as mean ± SEM. Differences were considered statistically significant when *p* < 0.05.

## 3 Results

### 3.1 Effects of nutritional status on brain-pituitary *lpxrfa* mRNA levels

**Figure 1:**
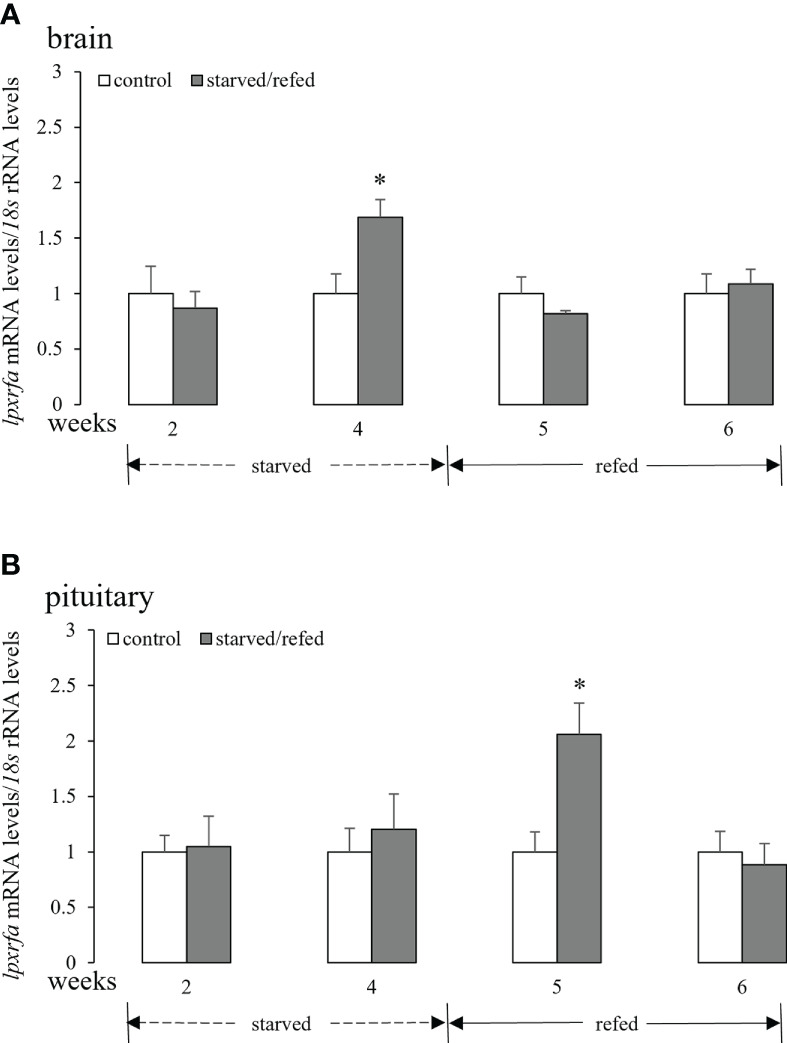
Effects of nutritional status on the brain **(A)** and pituitary **(B)** *lpxrfa* mRNA levels in half-smooth tongue sole. Data were normalized against *18s* transcripts and are presented as mean ± SEM (n = 4). A star indicates significant difference between fed and starved/refed groups (*p* < 0.05).

As shown in **Figure 1A**, there was no significant changes in the brain *lpxrfa* mRNA levels after 2 weeks of food deprivation when compared to the control group. However, evident increase in the brain *lpxrfa* expression was observed after 4 weeks of starvation (**Figure 1A**). The brain *lpxrfa* mRNA levels of starved fish were not significantly different from the corresponding controls during the refeeding period (**Figure 1A**). The pituitary *lpxrfa* gene in fish that fasted for 2-4 weeks displayed similar expression profiles compared with normal fed animals (**Figure 1B**). The pituitary *lpxrfa* transcripts displayed an evident increase at 5 weeks (1 week after refeeding), and dropped back to the levels of the control fish at 6 weeks (**Figure 1B**).

### 3.2 Effects of nutritional status on brain-pituitary *lpxrfa-r* mRNA levels

**Figure 2:**
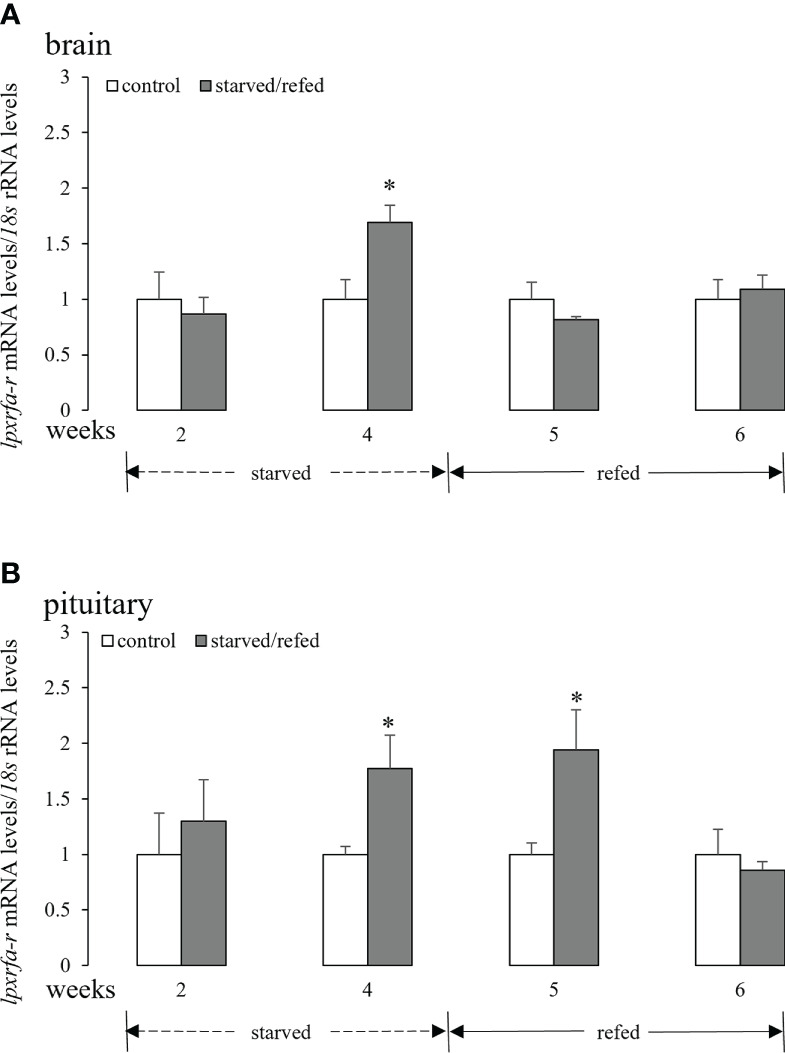
Effects of nutritional status on the brain **(A)** and pituitary **(B)** *lpxrfa-r* mRNA levels in half-smooth tongue sole. Data were normalized against *18s* transcripts and are presented as mean ± SEM (n = 4). A star indicates significant difference between fed and starved/refed groups (*p* < 0.05).

Fasting for 4 weeks promoted brain *lpxrfa-r* mRNA levels, and brain *lpxrfa-r* expression returned to basal levels after refeeding for 1 week and 2 weeks (**Figure 2A**). In the pituitary (**Figure 2B**), *lpxrfa-r* mRNA levels increased markedly after starvation for 4 weeks, still keeping high at 5 weeks, and declined to the levels of control group at 6 weeks (2 weeks after refeeding).

### 3.3 Effects of nutritional status on brain-pituitary *kiss2* mRNA levels

**Figure 3:**
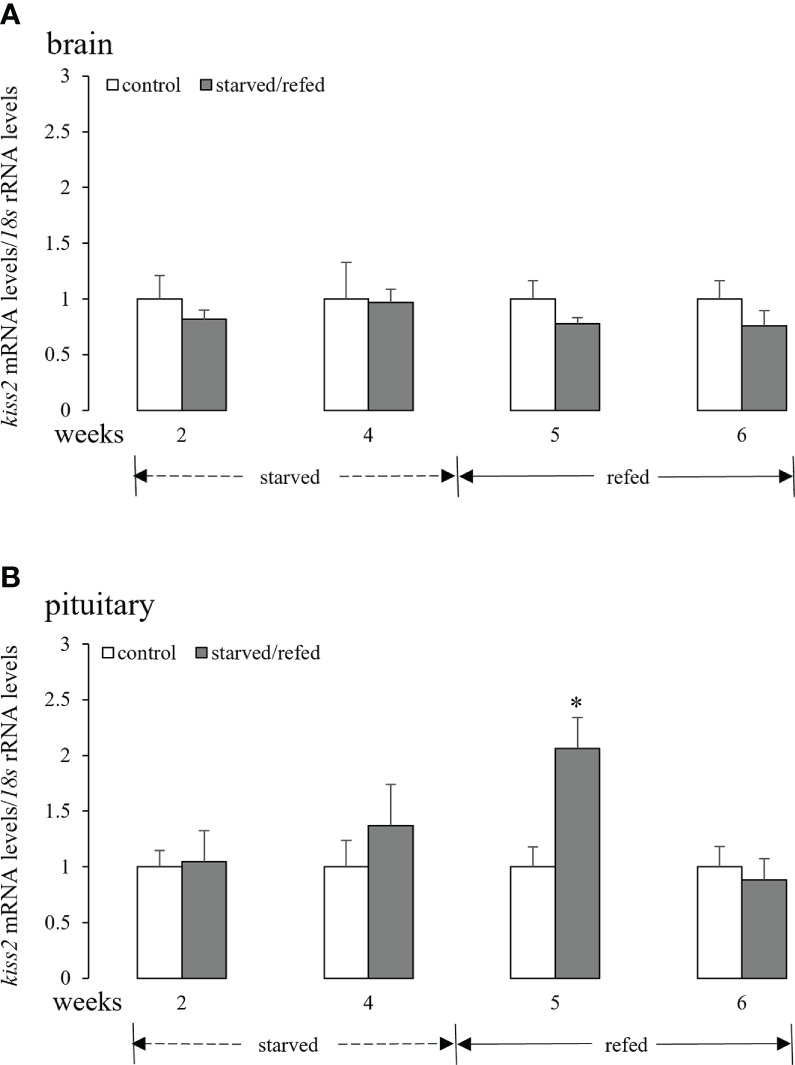
Effects of nutritional status on the brain **(A)** and pituitary **(B)** *kiss2* mRNA levels in half-smooth tongue sole. Data were normalized against *18s* transcripts and are presented as mean ± SEM (n = 4). A star indicates significant difference between fed and starved/refed groups (*p* < 0.05).

As shown in **Figure 3A**, neither food deprivation nor refeeding altered brain *kiss2* mRNA levels. Similarly, pituitary *kiss2* mRNA levels did not show any significant changes after fasting for 2 weeks or 4 weeks. However, pituitary *kiss2* transcripts increased markedly after 1 week of refeeding, and there were no significant differences between the two groups after 2 weeks of refeeding (**Figure 3B**).

### 3.4 Effects of nutritional status on brain-pituitary *kiss2r* mRNA levels

**Figure 4:**
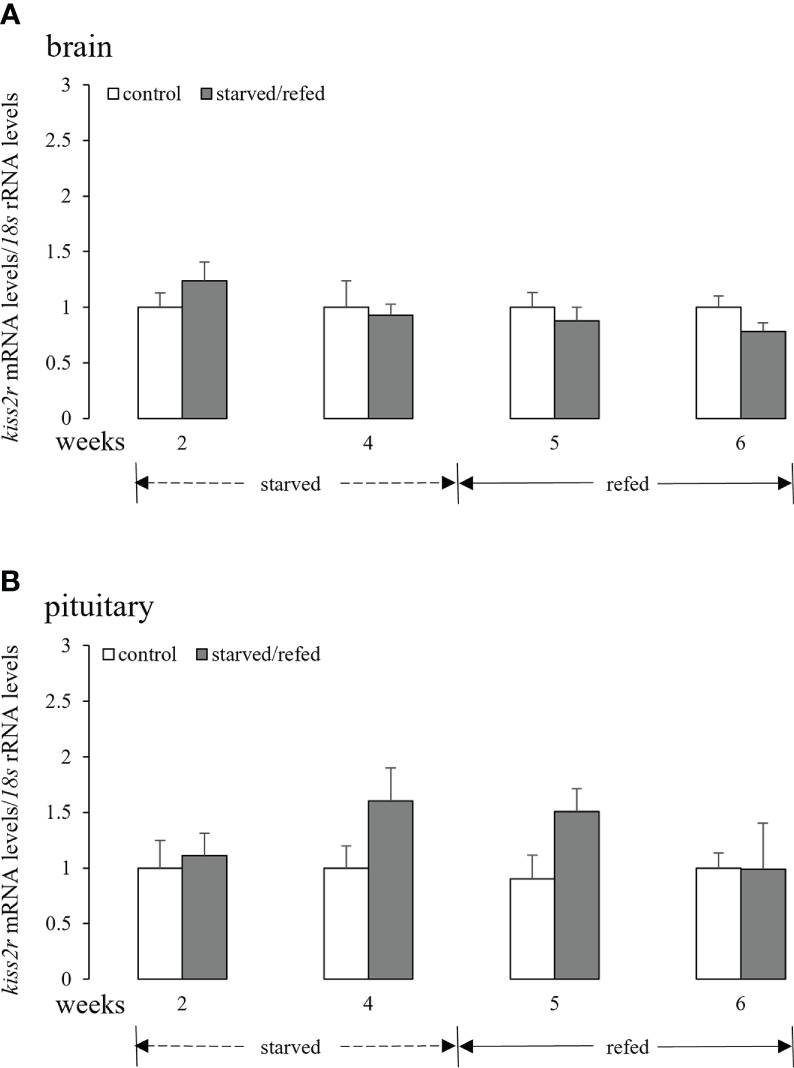
Effects of nutritional status on the brain **(A)** and pituitary **(B)** *kiss2r* mRNA levels in half-smooth tongue sole. Data were normalized against *18s* transcripts and are presented as mean ± SEM (n = 4). A star indicates significant difference between fed and starved/refed groups (*p* < 0.05).

No apparent differences between the two groups in the expression of brain *kiss2r* were observed during the starvation and refeeding periods (**Figure 4A**). Similar results were obtained for pituitary *kiss2r* transcripts, although a tendency of increase was noticed at 4 weeks and 5 weeks with their mean values not statistically different from the corresponding controls (**Figure 4B**).

## 4 Discussion

Reproduction is tightly coupled to metabolic status, and food restriction disturbs the reproductive axis by altering the signaling of some hormones or neuropeptides (34, 58, 59). It has been well demonstrated that both LPXRFa and Kiss peptides play a key role in the regulation of reproductive axis at multiple levels in vertebrates, including fish. However, the link between energy balance and reproduction *via* these two neuropeptides is still largely unknown (2, 26, 27, 52, 60). In the current study, we evaluated the effects of nutritional status on the transcript levels of the elements of both LPXRFa/LPXRFa-R and Kiss2/Kiss2R systems at the brain-pituitary levels of half-smooth tongue sole.

Our results showed that starvation stimulated mRNA levels of *lpxrfa* and *lpxrfa-r* in the brain along with pituitary *lpxrfa-r*, with no effects on pituitary *lpxrfa* expression in half-smooth tongue sole. Interestingly, fasting increased the brain *lpxrfa* transcripts in wild-type zebrafish females, but not in males (47). However, brain *lpxrfa* expression was higher in fasted Casper zebrafish males, but not in females (47). To our knowledge, brain and pituitary *lpxrfa-r* expression has not been investigated in fish under fasting conditions. Increased brain *lpxrfa* expression levels or LPXRFa-immunoreactive cell number in response to food deprivation was also observed in other species, including chicks (43, 61), Pekin ducks (36) and Syrian hamsters (62). Considering the orexigenic role of LPXRFa in birds and mammals (63), it is reasonable to assume that elevation of LPXRFa in the brain under fasting condition is sufficient to induce feeding behavior. Fasting did not alter *lpxrfa* expression in the hypothalamus of zebra finch males, in both mRNA and immunoreactivity levels (45). However, the number of LPXRFa-immunoreactive cells declined significantly in zebra finch females, showing sexual dimorphism of LPXRFa changes in response to nutritional stress (44). In addition, hypothalamic *lpxrfa-r* expression was lower in fasted chicks, perhaps due to receptor down-regulation in response to increased *lpxrfa* expression (61). Taken together, complex regulation of the LPXRFa/LPXRFa-R system exists in various species during negative metabolic state.

Similarly, the actions of negative nutritional status on the Kiss/KissR system are controversial. In the current study, neither *kiss2* nor *kiss2r* were altered in the brain and pituitary after starvation for 2 or 4 weeks, although an evident increase in pituitary *kiss2* expression was observed at the first week after refeeding. In another flatfish species, the Senegalese sole, fasting increased mRNA levels of *kiss2* and *kiss2r* in the hypothalamus, without any effects in the stomach (50). Interestingly, in wild-type zebrafish, fasting increased the brain expressions of *kiss1* in females and *kiss2* in males, respectively (47). However, neither *kiss1* nor *kiss2* transcripts were affected by fasting in Casper zebrafish (47). In male European sea bass, hypothalamic *kiss1*, *kiss2*, *kiss1r* and *kiss2r* transcripts were elevated after a prolonged period of food restriction (64). Food deprivation also resulted in a significant increase in hypothalamic *kiss2* and *kissr2_v1* mRNA levels in adult pejerrey males, without affecting *kissr2_v1* and *kissr2_v2* expression in the testis and habenula (51). By contrast, starvation decreased *kiss1* and *kissr1* expression in the hypothalamus of rhesus monkeys and mice (48, 65). Fasting also reduced brain *kiss1* expression in rats (66), lambs (67), and monkeys (68). Interestingly, a decrease in hypothalamic *kiss1* with a concomitant rise in *kissr1* mRNA levels was noticed in fasted rats (49). Altogether, kisspeptin signaling also mediates energy balance effects on the reproductive axis in fish, but the neuroendocrine mechanisms underlying the actions of undernutrition and low energy availability on the reproductive axis may differ between mammals and teleosts (69).

In summary, food deprivation differentially modulates gene expression of the components of LPXRFa and Kiss systems in half-smooth tongue sole. Combined with results from previous studies, differences of LPXRFa and Kiss in response to starvation could occur because of variations in species, sex, reproductive status, tissue and the elapsed time after treatment, indicating that LPXRFa and Kiss may provide a molecular switch between reproduction and appetite in vertebrates. The nature of starvation-elicited metabolic signals that alter LPXRFa and Kiss signaling is yet not well known, especially in teleosts (34, 59), and further studies are urgently needed to clarify how multiple signals work in concert to control reproduction during negative energy balance.

## Data availability statement

The original contributions presented in the study are included in the article/supplementary material, further inquiries can be directed to the corresponding author/s.

## Ethics statement

The animal study was reviewed and approved by Animal Care and Use Committee of Yellow Sea Fisheries Research Institute, Chinese Academy of Fishery Sciences.

## Author contributions

BW and YX designed this study. BW, AC, YZ and YJ performed the sampling of the fish. BW and AC conducted the RT-qPCR analysis and analyzed the data. BW wrote the first draft of the manuscript. YX and XL edited the manuscript. BW and YX provided funding. All authors contributed to the article and approved the submitted version.
